# Effect of screw thread type on implant-related complications following femoral neck fracture fixation in non-geriatric adults: a multicenter, retrospective cohort study

**DOI:** 10.3389/fsurg.2025.1745105

**Published:** 2026-01-13

**Authors:** Yuquan Bian, Kai Yang, Sen Lin, Shizan He, Dajun Jiang, Weitao Jia

**Affiliations:** 1 Department of Orthopedic Surgery, Shanghai Sixth People’s Hospital Affiliated to Shanghai Jiao Tong University School of Medicine, Shanghai, China; 2 Department of Radiology, Shanghai Sixth People’s Hospital, Shanghai, China; 3 Postgraduate Training Base of Jinzhou Medical University, Shanghai Sixth People’s Hospital, Jinzhou Medical University, Jinzhou, China

**Keywords:** comminuted fractures, femoral neck fractures, fully threaded screws, partially threaded screws, propensity score matched

## Abstract

**Introduction:**

This study compared implant-related complications after femoral neck fracture fixation using partially vs. fully threaded cannulated screws in non-geriatric patients.

**Methods:**

This retrospective cohort study included 1,035 patients with femoral neck fractures aged 18–60 years treated with cannulated screws from 2012 to 2022. Patients were followed until clinical failure—defined as the occurrence of any of the following: implant cut-out, femoral neck shortening, nonunion, or avascular necrosis—or for a minimum of 2 years. Patients were categorized into two groups according to thread type and balanced using propensity score matching. The primary outcome was the clinical failure rate. Subgroup analyses were performed for comminuted vs. non-comminuted fractures.

**Results:**

The overall clinical failure rate was 35.4%, including implant cut-out (1.9%), femoral neck shortening (15.0%), nonunion (9.3%) and avascular necrosis (23.5%); this rate was significantly higher for those with comminuted fractures. After balancing covariates, the partially threaded group demonstrated higher rates of femoral neck shortening (13.7% vs. 6%, *p* = 0.017) and avascular necrosis (17.9% vs. 9.5%, *p* = 0.027) than the fully threaded group in the overall cohort. In the non-comminuted subgroup, the partially threaded group was associated with significantly lower rates of implant cut-out (0% vs. 8.7%, *p* < 0.01) than the fully threaded group. However, in the comminuted fracture subgroup, the fully threaded group exhibited significantly lower incidences of femoral neck shortening (6.6% vs. 22.4%, *p* < 0.01) and avascular necrosis (10.5% vs. 36.2%, *p* < 0.01).

**Conclusions:**

Fracture comminution increases the risk of femoral neck shortening and avascular necrosis. In non-comminuted fractures, the use of partially threaded screws should be prioritized. However, in comminuted fractures, using fully threaded screws decreases rates of femoral neck shortening and avascular necrosis. This study provides evidence to guide cannulated screw thread type selection for comminuted fractures and paves the way for future randomized controlled trials.

## Introduction

Femoral neck fractures (FNFs) in patients aged <60 years remain challenging (1, 2) because treatment prioritizes preservation of the native femoral head and avoidance of early arthroplasty. However, FNFs are associated with fixation failure rates as high as 41.9% and avascular necrosis (AVN) rates ranging from 16% to 21% (3). Additionally, 24.3% of patients require subsequent reconstructive surgery (4), resulting in a 43.2% rate of permanent disability within 2 years, increasing medical care costs as well as social and economic pressure (4–6). Therefore, selecting the most appropriate implant is crucial for restoring fracture stability, facilitating revascularization and improving clinical outcomes (7).

Currently, cannulated screws remain a classic and widely used internal fixation in femoral neck fracture (8) because they are less invasive with less blood loss and cost-effective (9). Traditionally, partially threaded screws have been preferred because they enable continuous dynamic compression and facilitate fracture healing (10). However, excessive interfragmentary compression may be associated with severe femoral neck shortening (11–13). Conversely, fully threaded screws offer the advantage of length-stable fixation. Biomechanical studies have revealed that fully threaded screws offer superior resistance to shear forces (14) and fracture collapse (15) in femoral neck fractures, leading to improved stiffness, reduced severe femoral neck shortening, and improved mechanical stability (16, 17). While some studies have suggested that fully threaded screws may increase the risk of nonunion or even severe complications such as intra-pelvic penetration (18, 19), this remains the primary concern among clinicians, but high-quality clinical studies are lacking. Therefore, clinical data from large cohorts and evidence-based guidance for clinical applications are urgently needed.

A retrospective cohort study was conducted using a national, multicenter database to compare the complication rates between fully threaded and partially threaded screws, with a focus on their performance in comminuted fractures.

## Patients and methods

### Patient enrolment

**Figure 1:**
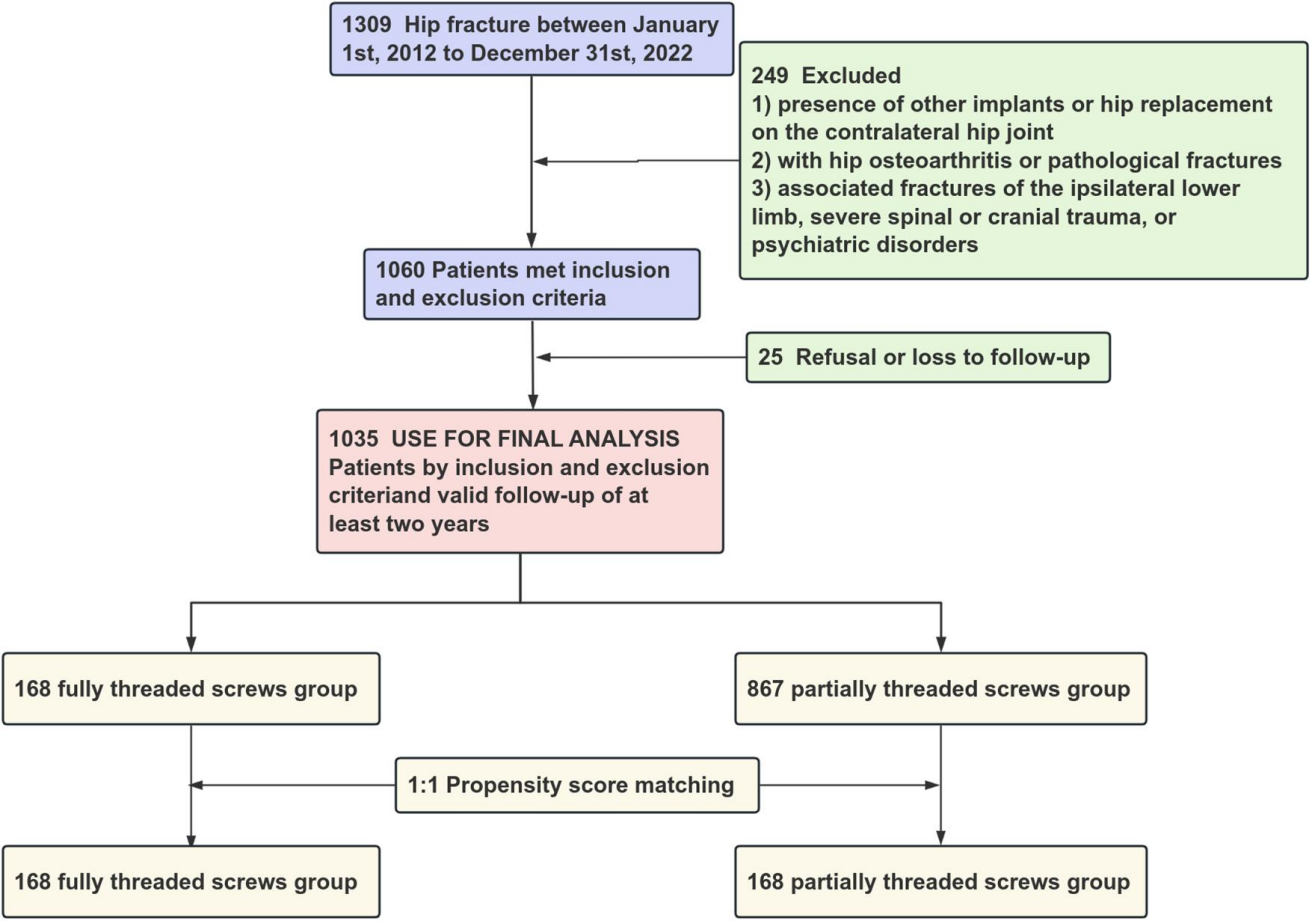
Flow diagram for patient selection process.

A retrospective review was conducted on patients with FNFs treated between January 2012 and December 2022 across three medical centers, including the largest center in China. To reduce confounding effects from the baseline covariates, a 1:1 propensity score matching (PSM) was performed, as shown in Figure 1.

This study was conducted in accordance with the ethical principles set forth in the Declaration of Helsinki. The study protocol was reviewed by the Ethics Committee of Shanghai Sixth People's Hospital [Shanghai, China; approval number: 2022-KY-059 (K)] and successfully registered with the China Clinical Trial Center (clinical trial registration number: ChiCTR2200059592).

### Patient selection criteria

The inclusion criteria were as follows: (1) patients (aged 18–60 years) with a history of trauma, (2) diagnosed with FNFs, and (3) treated with internal fixation using three fully or partially threaded screws. The exclusion criteria were as follows: (1) presence of other implants or hip replacement on the contralateral hip joint; (2) with hip osteoarthritis or pathological fractures; (3) associated fractures of the ipsilateral lower limb, severe spinal or cranial trauma, or psychiatric disorders.

### Surgical technique

Following the injury, preoperative examination including radiographic data was collected and surgery was carried out for all patients diagnosed with FNFs. Closed reduction was attempted first. If the closed reduction failed, open reduction was performed. To ensure adequate surgical quality, all surgeries were performed under general anesthesia by three senior orthopedic trauma surgeons. After achieving acceptable reduction, three 6.5 mm partially threaded or fully threaded screws were placed in either a triangular or inverted triangular configuration. In some surgeries using partially threaded screws, washers were used according to the surgeon's preference. All screws were identical in diameter and material composition. The reduction quality was evaluated using the Garden index and Lowell's S-patterns. Implant positioning was evaluated using the IMPO scoring system (20). Patients were routinely scheduled for standardized clinical and radiographic assessments at 6 weeks, 3 months, 6 months, 12 months, and annually thereafter (including 24 months). Follow-up data were obtained from outpatient charts and imaging archives. If a patient missed a scheduled visit, our clinic contacted them by telephone to reschedule to obtain updated radiographs. Patients were advised to avoid weight-bearing activities during the initial 6 weeks. Gradual partial weight bearing was introduced only if radiographic assessments indicated satisfactory bone union. Fracture union was assessed using both radiographic and clinical criteria. Radiographic union was defined as a Radiographic Union Score for Hip (RUSH) of ≥18 (21). The RUSH score comprises four components: cortical bridging, disappearance of the cortical fracture line, trabecular consolidation, and disappearance of the trabecular fracture line. Clinical union was defined by the 3 min walk test (3MWT) as the ability to ambulate continuously for 3 min with a total step count of ≥30.

### Measurements

Patient demographic variables included age, sex, body mass index (BMI), tobacco use (defined as individuals who had smoked at least 100 cigarettes in their lifetime and who maintained a habit of smoking one cigarette per day for a minimum of 6 months) (22), hypertension, and diabetes mellitus. Fracture displacement was characterized by the Garden Type (non-displaced: Garden Types I and II; displaced: Garden Types III and IV) and the Pauwels classification (Pauwels I-II: Pauwels Angle ≤ 50°; Pauwels III: Pauwels Angle > 50°) (23, 24). Bone quality was assessed using the Singh index, which grades proximal femur osteoporosis into six levels. A Singh index of ≤4 strongly suggests osteoporosis (25, 26). Comminution was assessed using preoperative computed tomography (CT). A fracture was considered comminuted if separate fragments >1 cm were present in any dimension that were not connected to the head-neck or shaft-neck fragments (27). The surgical variables included time to surgery, and reduction quality (good or poor). Reduction quality was assessed using the Garden index based on two angles: first, on the anteroposterior view, the angle between the central axis of the medial trabeculae in the capital fragment and the medial cortex of the femoral shaft; and second, on the lateral view, the angle between the central axis of the femoral head and the central axis of the femoral neck. Garden index was graded into four categories: anatomic (AP 160°, lateral 180°), acceptable (AP/lateral 155°–180°), borderline acceptable (AP/lateral 150°–155° or >180°), and unacceptable (AP <150° or >185°). Reduction quality was recorded as a binary variable: good (anatomic or acceptable) vs. poor (borderline acceptable or unacceptable) (28), while also requiring the presence of intact Lowell's S-patterns (“lazy S”), defined as continuous anterior and posterior cortical lines without gaps or steps in both views (29). The IMPO scoring system, which assesses implant positioning, consists of 6 items. An IMPO score less than 5 is associated with an increased risk of reoperation (20). All radiographic measurements were independently evaluated by two senior surgeons (JD and YB) according to the methods described above. If a dispute existed, the other senior surgeon (JW) made the final determination. Interobserver reliability was evaluated using the intraclass correlation coefficient (ICC 2,1), with ICC values ranging from 0.809–0.934. These parameters (age, sex, BMI, tobacco use, hypertension, diabetes, Garden/Pauwels types, comminution, bone quality, time to surgery, IMPO score, and reduction quality) were selected based on evidence from the past decade. Prior studies have identified these variables as prognostic factors for outcomes after femoral neck fracture. All variables were therefore included in the PSM model as potential confounders to minimize confounding and enhance comparability.

### Clinical outcome

The primary outcome was clinical failure, defined as the occurrence of any of the following events: femoral neck shortening, nonunion, screw cut-out, AVN. Femoral neck shortening was measured by comparing the position of the contralateral hip radiograph with the image taken at the final follow-up as previously described by Zlowodski and Weil (30), with shortening >1 cm considered severe. Nonunion was assessed using the RUSH, with a score <18 at 6 months postoperatively defined as nonunion (21). AVN was defined as type 2b or higher using Ficat's radiographic criteria (31). Screw cut-out was defined as projection of the screw from the femoral head into the acetabulum on radiographs (32).

### Statistical analysis

PSM was performed based on independent variables including sex, age, BMI, smoking, hypertension, diabetes, Garden type, Pauwels type, comminution, time from injury to surgery, bone quality, reduction quality and IMPO score. Propensity scores were estimated using a multivariable logistic regression model adjusted for covariates, and the dependent variable was screw thread type (fully vs. partially threaded). A 1:1 greedy matching without replacement was performed using a caliper width of 0.1 standard deviations (SD) of the logit of the propensity score (33, 34). A standardized mean difference (SMD) cutoff of 0.10 (35) was then adopted to assess the balance of baseline covariates between the fully threaded and partially threaded groups in the overall and matched cohorts.

Subgroup analyses were performed stratified by fracture comminution in the cohorts. Given that 1:1 PSM was insufficient to effectively eliminate baseline differences (SMD > 0.1), 1:2 PSM was performed in subgroup analyses. Interaction effects between thread types and outcomes were explored using logistic regression. Univariate analysis was used to compare baseline characteristics and complications between the two groups. Categorical variables were expressed as frequencies (percentages) and were compared using the chi-square test. Continuous parametric variables are represented by means (±SD) and were compared using t-tests, while nonparametric continuous variables are represented by median (IQR) and were compared using the Mann–Whitney test. Statistical significance was set at *p* < 0.05. All tests were two-sided. All statistical analyses were performed using IBM SPSS Statistics (version 29.0), and PSM was performed in R (version 4.4.1; R Foundation for Statistical Computing). All patients received education about the purpose of the study and provided informed consent to participate.

## Results

A total of 1,035 eligible patients with FNFs were finally included in our cohort. Of these, 867 (83.77%) and 168 (16.23%) were treated with partially and fully threaded screws, with a mean age of 48.7 years. These patients underwent an average follow-up of 33.2 ± 8.4 months (range: 3–60 months). Among them, 573 (55.3%) presented with displaced fractures, and 399 (38.5%) presented with comminution (Supplementary Table S1). Washer use was not statistically different between patients with and without clinical failure in partially threaded screws (32.0% vs. 31.2%, *p* = 0.91). The overall clinical failure rate was 35.4%. Event-specific rates were 1.9% for implant cut-out, 15.0% for femoral neck shortening, 9.3% for nonunion, and 23.5% for AVN; complications were not mutually exclusive. Respectively, comminuted fractures showed a higher failure rate than non-comminuted fractures (49.8% vs. 26.4%, *p* < 0.01). Significant differences were observed between the two groups in terms of age, sex, Garden classification, Pauwels classification, diabetes and time to surgery. After propensity score matching, baseline variables were well balanced (*p* > 0.05). All covariates included in the PSM had SMD values <0.10 after matching, indicating negligible differences between the groups (Table 1).

**Table 1:** Baseline data of indicators and clinical data.

Variables	Fully threaded (*N* = 168)	Partially threaded (*N* = 168)	*P* value	SMD
Age, years	50 (37–56)	53 (42.25–55)	0.613	−0.03
Male sex	98 (58.3%)	99 (58.9%)	0.912	−0.01
BMI, kg/m^2^	22.58 (21.63–23.75)	23.18 (22.12–23.97)	0.842	−0.06
Tobacco use	48 (28.6%)	52 (31%)	0.634	−0.05
Garden type		0.444	0.08	
Nondisplaced (Garden I II)	94 (56%)	87 (51.8%)		
Displaced (Garden III IV)	74 (44%)	81 (48.2%)		
Pauwels type			0.822	0.03
Pauwels I II	105 (62.5%)	103 (61.3%)		
Pauwels III	63 (37.5%)	65 (38.7%)		
Bone quality		0.584	0.06	
Poor	89 (53%)	94 (56%)		
Good	79 (47%)	74 (44%)		
Time to surgery, days			0.289	0.03
<24 h	33 (19.6%)	41 (24.4%)		
24–48 h	83 (49.4%)	81 (48.2%)		
>48 h	52 (31%)	46 (27.4%)		
Comorbidity				
HTN	15 (8.9%)	15 (8.9%)	1	0.00
DM	19 (11.3%)	22 (13.1%)	0.618	−0.06
Reduction quality			0.725	0.04
Good	149 (88.7%)	151 (89.9%)		
Poor	19 (11.3%)	17 (10.1%)		
IMPO scoring system			1	0.00
≤4 point	78 (46.4%)	78 (46.4%)		
5–6 point	90 (53.6%)	90 (53.6%)		
Configuration			0.150	0.17
Triangular	69 (41.1%)	83 (49.4%)		
Inverted triangular	99 (58.9%)	85 (50.6%)		

SMD, standardized mean difference; IMPO, implant positioning; propensity score matching was utilized for matching.

**Figure 2:**
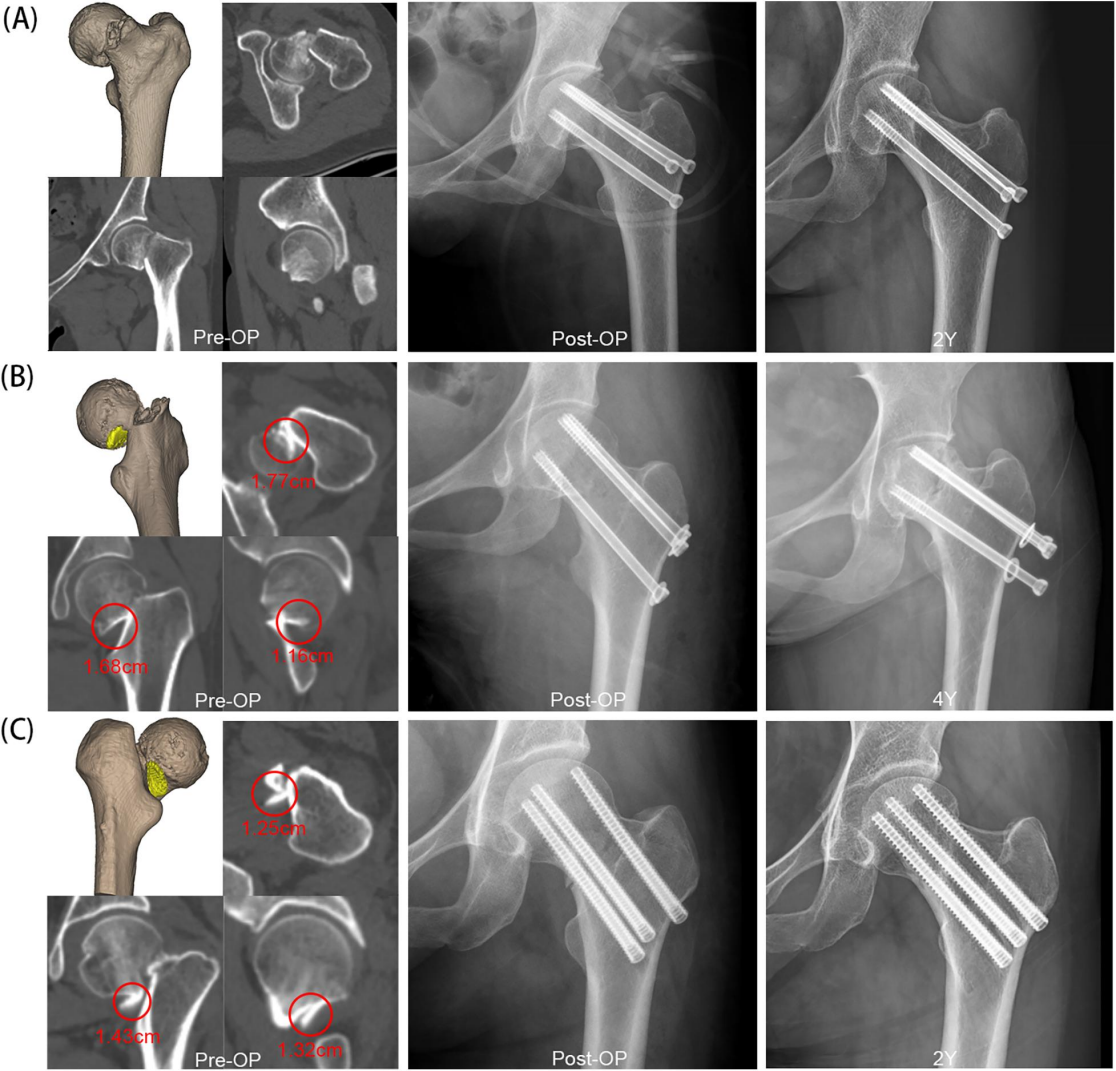
Preoperative and postoperative radiographs of femoral neck fractures treated with three cannulated screws. The fragmented debris are circled, with their maximum diameter indicated. **(A)** A 50-year-old woman with a non-comminuted femoral neck fracture exhibited good radiographic healing two years after fixation with partially threaded screw fixation; **(B)** A 34-year-old woman with a comminuted femoral neck fracture developed severe femoral neck shortening and avascular necrosis of the femoral head four years after undergoing partially threaded screw fixation; **(C)** A 53-year-old woman with a comminuted femoral neck fracture exhibited good radiographic healing two years after fixation with fully threaded screws.

First, 1:1 PSM adjustment was performed for patients treated with partially threaded and fully threaded screws. After PSM adjustment, the fully threaded (*n* = 168) and partially threaded (*n* = 168) groups were well balanced at baseline (*p* > 0.05). The partially threaded screw group had a lower incidence of implant cut-out (0.6% vs. 7.7%, *p* < 0.01). In contrast, the fully threaded screw group demonstrated lower rates of femoral neck shortening (6% vs. 13.7%, *p* = 0.017) and avascular necrosis (9.5% vs. 17.9%, *p* = 0.027) (Table 2). Significantly higher failure rate was observed in patients with comminuted fractures than in those without comminution (49.8% vs. 26.4%, *p* < 0.01). Accordingly, patients were stratified into subgroups based on comminution and repeated PSM adjustment for subgroup analyses. In the non-comminuted subgroup (*n* = 276), no significant differences were observed in femoral neck shortening (5.4% vs. 5.4%, *p* = 1) or avascular necrosis rates (8.7% vs. 10.3%, *p* = 0.668) between the two groups. However, fully threaded screws were associated with significantly higher rates of nonunion (8.7% vs. 2.7%, *p* = 0.027) and cut-out (8.7% vs. 0%, *p* < 0.01) than partially threaded screws (Table 3). Conversely, In the comminuted fracture subgroup (*n* = 228), the fully threaded screw group demonstrated significantly lower incidence of femoral neck shortening (6.6% vs. 22.4%, *p* < 0.01) and avascular necrosis (10.5% vs. 36.2%, *p* < 0.01) than the partially threaded screw group. No significant differences were observed between the two groups in nonunion (19.7% vs. 13.8%, *p* = 0.249) or cut-out rates (6.6% vs. 3.3%, *p* = 0.254) (Table 3) (Figure 2).

**Table 2:** Outcomes after PSM matching.

Outcomes	Fully threaded (*n* = 168)	Partially threaded (*N* = 168)	Crude OR (95% CI)	*P* value
Clinical failure	55 (32.7%)	53 (31.5%)	1.06 (0.67–1.67)	0.816
Femoral neck shortening	10 (6%)	23 (13.7%)	0.40 (0.18–0.87)	**0** **.** **017**
Avascular necrosis	16 (9.5%)	30 (17.9%)	0.48 (0.25–0.93)	**0** **.** **027**
Nonunion	23 (13.7%)	14 (8.3%)	1.75 (0.87–3.52)	0.117
Cut-out	13 (7.7%)	1 (0.6%)	14.01 (1.81–108.33)	***p* < 0** **.** **01**

Bold text indicates statistically significant result; PSM, propensity score matching.

**Table 3:** Outcomes after PSM matching in subgroup.

Outcomes	Non-comminuted fracture population				Comminuted fracture population			
	Fully threaded (*N* = 92)	Partially threaded (*N* = 184)	Crude OR (95% CI)	*P* value	Fully threaded (*N* = 76)	Partially threaded (*N* = 152)	Crude OR (95%CI)	*P* value
Clinical failure	24 (26.1%)	32 (17.4%)	1.68 (0.92–3.06)	0.091	31 (40.8%)	75 (49.3%)	0.71 (0.41–1.24)	0.223
Femoral neck shortening	5 (5.4%)	10 (5.4%)	1 (0.33–3.02)	1	5 (6.6%)	34 (22.4%)	0.24 (0.09–0.65)	***p* < 0** **.** **01**
Avascular necrosis	8 (8.7%)	19 (10.3%)	0.83 (0.348–1.97)	0.668	8 (10.5%)	55 (36.2%)	0.21 (0.09–0.46)	***p* < 0** **.** **01**
Nonunion	8 (8.7%)	5 (2.7%)	3.41 (1.08–10.74)	**0** **.** **027**	15 (19.7%)	21 (13.8%)	1.53 (0.74–3.18)	0.249
Cut-out	8 (8.7%)	0 (0%)	/	***p* < 0** **.** **01**	5 (6.6%)	5 (3.3%)	2.07(0.58–7.38)	0.254

Bold text indicates statistically significant result; PSM, propensity score matching.

## Discussion

This study conducted a comparative analysis of two commonly used cannulated screw types, evaluating their therapeutic effects on femoral neck fractures. In the overall cohort, partially threaded screws exhibited a lower nonunion and cut-out rate, whereas fully threaded screws were associated with less femoral neck shortening and AVN. In non-comminuted femoral neck fractures, the use of partially threaded screws reduces the risk of cut-out and nonunion. In comminuted femoral neck fractures, fully threaded screws significantly decreased femoral neck shortening and AVN, without substantial increases in screw cut-out or nonunion. These findings support the safety and efficacy of partially threaded screws in treating non-comminuted femoral neck fractures, while fully threaded screws demonstrate optimal performance in comminuted femoral neck fractures.

Partially threaded screws provide dynamic compression at the fracture site during weightbearing at the cost of reduced stability and femoral neck shortening. In contrast, dynamic compression is absent with fully threaded screws. Although compressive forces can promote fracture healing according to stress–strain theory (36), it remains unclear whether dynamic compression is necessary or if intraoperative initial compression suffices. Frandsen's study has reported higher failure rates for displaced femoral neck fractures when sliding hip screws were used in a dynamic compression mode compared with static locking (37). Similarly, inappropriate use of partially threaded screws in certain fracture patterns may cause excessive dynamic compression and result in severe femoral neck shortening. Studies have revealed that >10 mm of shortening substantially reduces the abductor moment arm, leading to abductor muscle dysfunction and Trendelenburg gait (12). In addition, screw back-out following severe femoral neck shortening can result in soft-tissue irritation and chronic hip pain, collectively contributing to lower Harris Hip and SF-36 scores (38).

Fracture comminution is common in high-energy fractures (3, 39) and occurs in up to 49.4% of FNFs (40). The limited contact at the fracture ends may result in a loss of reduction and potential rotational instability (41), resulting in a bony buttress defect that complicates anatomical reduction and increases the likelihood of fracture re-displacement under physiological loads. In comminuted fractures, the use of partially threaded screws may induce excessive femoral neck shortening and associated with higher risks of revision arthroplasty (12). By contrast, fully threaded screws have been shown in previous biomechanical studies to provide superior resistance to shear forces (42) and fracture collapse (15–17), which can prevent excessive dynamic compression in comminuted fractures and significantly reduce excessive femoral neck shortening (13). Although fully threaded screws showed a higher incidence of cut-out and nonunion in comminuted fractures, no significant differences were observed between two groups in our study. Therefore, fully threaded screw fixation is an effective and relatively safe option for the treatment of comminuted fractures.

In our original cohort, fully threaded screws were more frequently utilized in younger patients and those with stable, non-displaced femoral neck fractures, potentially introducing selection bias and confounding outcomes. To mitigate this, propensity score matching was employed, a robust method that mimics randomization in retrospective studies, thereby enhancing causal inference validity (43). Compared to traditional regression—which risks over- or underestimating effects with limited confounders common in orthopedic cohorts—PSM provides a nonparametric alternative that does not rely on distributional assumptions and enables precise marginal treatment effect estimation at the population level. By balancing baseline covariates, PSM minimized systematic differences, optimized bias control over exact matching, and maintained adequate sample size for statistical power, strengthening our study's methodological rigor and ensuring outcomes reflect the intervention rather than unmeasured biases.

Our study had several limitations. First, functional outcome scores were not included. Future research is warranted to further elucidate the association between screw thread types and functional outcome scores. Second, the retrospective nature of this study had inherent limitations. The lack of randomization indicates that unmeasured confounding factors may have influenced the outcomes. Although this study revealed an association between the screw type and clinical prognosis, causality cannot be definitively established.

## Conclusion

Femoral neck fractures in non-geriatric adults are associated with high fixation failure, reoperation and disability rates, and optimal choice of cannulated screw thread types remains controversial. This study is the first to analyze a large-cohort clinical dataset on this topic to our knowledge. We found that partially threaded screws were associated with greater femoral neck shortening and a higher rate of avascular necrosis (AVN). In contrast, partially threaded screws showed lower rates of nonunion and implant cut-out than fully threaded screws in the overall cohort. In non-comminuted fractures, the use of partially threaded screws should be prioritized. However, in comminuted fractures, partially threaded screws are associated with increased femoral neck shortening and AVN. Fully threaded screws mitigate these complications without increasing cutout or nonunion, confirming their safety and efficacy in comminuted fractures. These findings will guide case-by-case decision-making and drive future randomized controlled trials.

## Data Availability

The raw data supporting the conclusions of this article will be made available by the authors, without undue reservation.
