# Supplementary material for: Effect of screw thread type on implant-related complications following femoral neck fracture fixation in non-geriatric adults: a multicenter, retrospective cohort study
Source: Front Surg. 2026 Jan 13;12:1745105. doi: 10.3389/fsurg.2025.1745105 (PMC12835346; doi:10.3389/fsurg.2025.1745105)
Supplement: Supplementary file 1 [file Table1.docx]

**Supplementary information to**

Effect of Screw Thread Type on Implant-Related Complications Following Femoral Neck Fracture Fixation in Non-geriatric Adults: A Multicenter, Retrospective Cohort Study

Yuquan Bian^1†^; Kai Yang^2†^; Sen Lin^1^; Shizan He^3^; Dajun Jiang^1*^; Weitao Jia^1*^.

|  | Fully thread (N=168) | Partially thread (N=867) | P value | SMD |
| --- | --- | --- | --- | --- |
| Age, years | 50(37-56) | 53(45-56) | **p<0.01** | 0.30 |
| Male sex | 98(58.3%) | 376(43.4%) | **p<0.01** | -0.30 |
| BMI, kg/m2 | 22.58(21.63-23.75) | 22.96(21.75-23.71) | 0.149 | 0.20 |
| Tobacco use | 48(28.6%) | 230(26.5%) | 0.584 | 0.05 |
| Garden type |  | | **p<0.01** | 0.27 |
| Nondisplaced (Garden I II) | 94(56%) | 368(42.4%) |  |  |
| Displaced (Garden III IV) | 74(44%) | 499(57.6%) |  |  |
| Pauwels type |  |  | 0.023 | 0.19 |
| Pauwels I II | 105(62.5%) | 459(52.9%) |  |  |
| Pauwels III | 63(37.5%) | 408(47.1%) |  |  |
| Bone quality |  | | 0.152 | 0.12 |
| Poor | 89(53%) | 511(58.9%) |  |  |
| Good | 79(47%) | 356(41.1%) |  |  |
| Time to surgery, days |  |  | **p<0.01** | 0.47 |
| <24h | 33(19.6%) | 75(8.7%) |  |  |
| 24-48h | 83(49.4%) | 346(39.9%) |  |  |
| 48h | 52(31%) | 446(51.4%) |  |  |
| Comorbidity |  |  |  |  |
| HTN | 15(8.9%) | 64(7.4%) | 0.490 | 0.06 |
| DM | 19(11.3%) | 152(17.5%) | 0.047 | -0.18 |
| Reduction quality |  |  | 0.859 | 0.01 |
| Good | 149(88.7%) | 773(89.2%) |  |  |
| Poor | 19(11.3%) | 94(10.8%) |  |  |
| IMPO scoring system |  |  | 0.239 | 0.09 |
| ≤4 point | 78(46.4%) | 360(41.5%) |  |  |
| 5-6 point | 90(53.6%) | 507(58.5%) |  |  |
| SMD = standardized mean difference; IMPO = Implant Positioning; propensity score matching was utilized for matching | | | | |

**Supplementary Table 1** Baseline data of indicators and clinical data before propensity score matching
